# The impact of globalization, renewable energy, and labor on sustainable development: A cross-country analysis

**DOI:** 10.1371/journal.pone.0315273

**Published:** 2025-02-21

**Authors:** Huynh Ngoc Chuong, Vo Tran Phuong Uyen, Nguyen Dang Phuong Ngan, Nguyen Thi Bao Tram, Nguyen Dao Mai Han, Pham Hoang Khanh Duyen

**Affiliations:** University of Economics and Law, HoChiMinh city, VIETNAM; Stellenbosch University, SOUTH AFRICA

## Abstract

Sustainable development stands as both a goal and a prevailing trend in the global economy all the time. However, a comprehensive understanding of the internal and external determinants influencing sustainable development is necessary for the formulation of appropriate policies and development strategies. This research investigates dimensions of sustainable development in the panel data of 104 selected countries from 2000 to 2020. These economies are categorized into four groups based on the level of development. The exclusive role is given to the impact of three key factors, based on the triple bottom line (TBL) model, such as globalization, labor, and renewable energy on sustainable development. We employ the panel unit root tests, cointegration tests, and pool mean group (PMG) approach to estimate the relationships between globalization, renewable energy, labor force, and sustainable development. The results indicate the positive effects of globalization, labor, and renewable energy on sustainable development. Furthermore, a higher level of renewable energy consumption promotes sustainable development within the divided groups. The findings highlight that the labor factor has a positive impact on the sustainable development of all groups of economies. Thereby, the sustainability policy are implied to focus on the educational policy, improving social stability and renewable energy sources, particularly in the middle trap countries.

## 1. Introduction

In recent years, there has been an increasing awareness and concern about pursuing sustainable practices in economic, environmental, and social spheres. The concept of sustainable development aims to maintain economic advancement and progress while protecting the long-term value of the environment. With recent stable management of traditional environmental issues such as water, air, and waste, especially in developed countries, the international community has proposed a concept of ‘sustainable development’, in which the community can respond to problems such as climate change risks and biodiversity losses while simultaneously achieving economic growth. Sustainable development has become a research area that has received a lot of attention in recent years. Researchers have debated the relative importance of ecological, economic, and social components and how to balance these dimensions. The human development index (HDI), which is an almost universally accepted measurement of the aspects of development, considers three pillars of human development: health, knowledge, and income.

According to the United Nations, SDGs stand for sustainable development goals, which represent a comprehensive set of aims and benchmarks that have been universally embraced by members of the UN to shape their agendas and policies. These goals, as stated by Olga and Janos in their study (2015) [1], are an expansion of the Millennium Development Goals (MDGs), encompassing 17 goals and 169 specific targets, addressing a wide range of challenges humanity faces. The SDGs endeavor to advance sustainable development in diverse aspects, including fostering economic growth, empowering women, and addressing environmental issues, etc [2].

The triple bottom line or 3Ps (profit, planet, and people), a framework developed by Elkington **[3]**, has come to be a globally significant approach in practices [4, 5]. The TBL was validated by integrating human well- being and the planet. Consequently, the economic dimension stands as a vital subsystem of sustainability, playing a pivotal role in navigating survival and evolution towards the future [6]. In this context, globalization emerges as the third pressure wave, evaluating the degree of sustainable economic development according to the TBL model, with a specific focus on the growing acknowledgment of sustainable development **[3]**. Globalization is a multifaceted development directly via social, economic, political, and cultural factors as well as has indirect implications via higher education. It is easier for nations to access resources such as capital, know-how, information, and green technologies, therefore it may have a positive influence on sustainable development. However, globalization can increase environmental degradation and ecological contamination, especially in lower-income countries [7]. Therefore, the net influence of globalization on sustainable development hinges on which factors are dominant [8].

Recent evaluations conducted by the Intergovernmental Panel on Climate Change (IPCC) and the Intergovernmental Science-Policy Platform on Biodiversity and Ecosystem Services (IPBES) have highlighted the threats posed to humanity due to the unsustainable use of natural resources. Thus far, land, freshwater, and ocean exploitation have been the chief causes of biodiversity loss. It is anticipated that climate change will increasingly become a significant additional factor driving the loss of biodiversity as well as households livelihood [9]. In this context, the TBL model suggested that renewable energy emerges as a viable environmental solution for sustainable development, addressing the management of finite biophysical resources and their reduction in processing [10]. Researchers argued that energy plays an important role in sustainable development [11, 12]. Improving the energy structure and promoting a clean energy transition has become the global consensus. Recent papers show that renewable energies are the most suitable energy sources for sustainable development [13, 14]. High-income countries have the proportion of renewable energy is 11.18% in total energy consumption. Meanwhile, the exploitation of renewable sources in developing countries is more urgent because of their rapid industrialization and urbanization. Due to the rising energy demand for growth, they have to accept carbon-industries with high emissions as part of their globalization and development [15, 16].

The relationships between renewable energy and globalization have been explored within the framework of economic development levels. The levels of economic growth may give rise to diverse interactive relationships in sustainable development [17, 18]. Güney demonstrated that high-income countries consider renewable energy a pivotal element in fostering sustainability within their territories [19]. Gasimli et al. underscored the significant and advantageous impacts of economic and political globalization on sustainable development, notwithstanding the decline in energy consumption within these nations and its influence on levels of globalization and sustainable development [20]. These interconnections can also be observed in other developed and developing nations. Hai et al. illustrated the significant differences in economic growth among countries, subsequently categorizing them into successful, trapped, or failed groups [21]. That implied the approaches to sustainable development led to the successful level of economic growth.

With a focus on inclusivity, it is evident that the SDGs require a deeper comprehension of development outcomes on the ground. Although most countries still use Gross Domestic Product (GDP) to assess standard of living and development, the HDI (Human Development Index) will do a better job than GDP of capturing what progress is being over a 15-year timeframe. In the meanwhile, researchers recognized the shortcomings of GDP, because it only measures a country’s productive capacity (the total value of goods and services in a country over time), not its overall well-being. A country’s education and health are regarded as equally important as its economic power. As a result, they developed the HDI, a summary metric that incorporates three key dimensions of human development: health, education, and living standards. Considering life expectancy and education along with per capita income in the HDI allow decision makers to flesh out a multifaceted aspect of human development and countries’ well-being progress. These relatively simple data points are essential indicators of social well- being and freedom, which provide a shared roadmap for the world to work towards committing sustainable development.

Sustainable development (SD) and globalization have been working against each other, resulting in a global state of poverty and inequality, putting a strain on the resources [22, 23]. Some argue that the primary challenge in making globalization and SD work is inequality, hindering the adoption of sustainable methods in developing countries. Others claim that the issue is rooted in the policies and regulations of global governance, which should be better regulated [24]. As a result, there is an urgent need for various approaches to address this issue, such as the UN’s sustainable development initiatives, involvement of citizens in global governance, and diverse frameworks for incorporating sustainable development into business practices.

One key motivation for choosing this topic stems from the need to understand how key factors influence sustainable development across different income levels and regions [25]. The importance of environmental policy and renewable energy is seen mitigating environmental degradation, emphasizing the relevance of examining these factors in a cross- country context [26]. By integrating insights from such studies and conducting a cross-country analysis encompassing various income groups, our present research aims to fill existing gaps and provide practical guidance for policymakers and stakeholders. Moreover, while there have been studies examining the individual effects of globalization, labor, and renewable energy on sustainable development, there is a need to bridge the gap by analyzing their interconnections and collective impact.

In this paper, the authors focus on the effects of human development, renewable energy, and globalization on SDG. We examine the relationship between development goals, renewable energy, and globalization in the sustainable framework. This paper follows the Triple Bottom Line (TBL) model to narrow down the “globalization” research scope into the economic dimension and focus mainly on how factors relevant to the economic sector affect sustainable development. In addition, the authors add the “success” levels in economic development over time. In this research paper, our practical context strengthens the focus on sustainable development goals across various economies, encompassing both developed and developing nations, spanning from low-income to high-income country levels. In consequence, we would provide many new arguments and perspectives when examining the diverse scopes of the sample panels. This research is expected to contribute to empirical results and implications for management practice in each country’s sustainable development policy.

This study is organized as follows. The first section gives details about the topic introduction. The second section includes the related hypothesis. The third section describes the proposed model and econometric approach, the fourth section explains the empirical results and discussion. The last section provides conclusions and implications.

## 2. Hypothesis development and proposed model

### 2.1 The effects of globalization on sustainable development

Within the TBL model, globalization is considered as the economic dimension [27], and in the context of globalization, growth occurs simultaneously in various fields, and the increasing interconnectedness demands a starting point for sustainable international policies [28]. This study also points out that globalization has a positive impact on sustainability in general and the increasing complexity of our global society means that sustainable development cannot be addressed from a singular national or disciplinary perspective. Moreover, while sustainable development in one aspect of globalization might not directly relate to sustainable growth in other areas, what is feasible on a national level may not be achievable on a global scale. Some evidence pointed out that globalization significantly impacts both short-term and long-term sustainable development [8]. The short-term effects of globalization on sustainable development can be attributed to external factors stemming from the internationalization of higher education institutions and increased access to financial resources, expertise, data, and eco-friendly technologies. Therefore, depending on the specific characteristics of each country, the long-term positive impacts of globalization on sustainable development may vary. Additionally, that the long-term results have verified that the causal link stems from economic globalization and leads to sustainable development.

The various components of globalization (economic, political, and social) have positive correlations with sustainable development [20]. Therefore, it is vital for countries integrating into the global economy and politics to pursue sustainable development. This positive impact on SDGs is evident through its contributions to economic growth, market access, knowledge sharing, cultural exchange, technological advancements, and global partnerships, all of which play a crucial role in promoting sustainable development and achieving the SDGs. Therefore, it is vital for countries integrating into the global economy and politics to pursue sustainable development. It is necessary to be aware that the impact of globalization on sustainable development is complex and multifaceted, requiring policymakers to implement diverse measures to mitigate negative effects and promote sustainable development, particularly in less developed regions and marginalized communities. Globalization can create opportunities for economic growth and development. It facilitates the exchange of ideas, technology, and resources across borders, which can help countries achieve certain SDGs, such as poverty reduction and economic growth. This interconnectedness can lead to improved access to markets and investment opportunities for developing nations [29–31].

However, globalization also hinders sustainable development and makes it infeasible, as highlighted by [32]. Globalization’s negative influence on sustainable development in the low-middle trap group is mentioned [33]. The study highlights that firms in the middle- income countries in Southeast Asia lack innovation, and globalization through trade and foreign direct investment (FDI) plays a significant role in addressing this issue. Additionally, Langnel and Pathranarakul argue that while globalization is likely to have a negative impact on sustainable development, this relationship may be moderated by the level of economic development and institutional quality [34]. In conclusion, globalization can have a negative influence on sustainable development, but the extent of this impact may vary depending on the quality of institutions, growth in the economy, and the stage of globalization.

Globalization holds promise for sustainability, especially in economies dominated by developed nations that focus on technological transformation [35]. However, it is important to note that the process and outcomes of globalization are inherently unequal and depend on local changes in economic, social, and political structures. Therefore, achieving a synchronized global transition towards sustainability is not feasible due to the persistent disparities in protection, regulation, compliance, and concerns at regional, national, and local levels. Nation-states exhibit varying attitudes towards economic globalization, as seen in their policy responses. These differences in policy responses reflect the diverse approaches taken by countries in addressing the challenges and opportunities presented by globalization. Some nation-states may embrace globalization by implementing policies that promote international trade and economic integration. In contrast, other countries may adopt protectionist measures to shield their economies from the perceived negative impacts of globalization. By analyzing the diverse policy responses of different countries, researchers and policymakers can gain insights into the complex dynamics of globalization and develop strategies to promote sustainable development in a globalized world.

***H1*: *The globalization of the economic dimension of TBL has a significant effect on sustainable development*.**

### 2.2 The effects of labor on sustainable development

The framework of sustainable development of the TBL model mentioned that labor emerges as a critical social factor, influencing the sustainable development of a nation [36]. The SDGs enhance the awareness of employees of the need to increase their productivity and create proactive citizens who can make a positive contribution to society [37]. Human resource development can be considered as one fundamental department/area to complete the goals at the organizational level. Therefore, the human element plays a dual role, as it is the initiator as well as the recipient in the accomplishment of SDGs. According to the observations of various business disciplines related to manufacturing and operational activities, the human factor is the primary source of the interconnection between HRM (Human Resource Management) and the SDGs, as the attitudes, behavior, and resource consumption of people have a direct influence on ecological and social practices. Moreover, striking an equilibrium between commercial growth, the protection of environmental resources, and the achievement of organizational targets is the primary objective of sustainable human resource management.

Dorward indicates how agricultural revolutions that raise agricultural labor productivity in poor agrarian economies can play multiple foundational roles in wider development processes [38, 39]. New technologies and resources that increase production per worker also increase food availability per worker. Higher labor productivity then lowers the cost (and hence price) of food relative to agricultural worker incomes, which raises agricultural workers’ budget surpluses after food expenditures and hence increases their real incomes. Research and policy for high rural labor productivity in sustainable and resilient agricultural and food systems therefore need much greater explicit attention in international policy than they have had in the past.

Most of the UN sustainable development goals are closely intertwined with migration and mobility. Empirical results explored how and to what extent labor migration from and to Central Asian countries influences the socio-economic aspects of the sustainable development of the region and countries [40, 41]. The movement of the workforce is a strong instrument of sustainable economic growth, higher living standards, more jobs, and, therefore, social stability in the countries. Thus, recognizing the importance of labor migration and receiving remittances the Governments of the Central Asian countries perceive them as tools for socio-economic growth and stability.

***H2*: *The labor of social dimension of TBL has a positive effect on sustainable development*.**

### 2.3 The effects of renewable energy on sustainable development

In the framework of sustainable development of the TBL model, renewable energy is determined as an environmental dimension [42]. From the perspective of high-income nations, renewable energy takes on a central role in fostering sustainable development within these countries [19]. Concurrently, this research substantiates a statistically significant negative impact of non-renewable energy consumption on the prospects of long-term sustainable development. Therefore, it is imperative for countries not only to increase their adoption of renewable energy but also to curtail the use of non- renewable energy resources to attain their lasting sustainability goals. In addition, energy is a critical element in controversies on sustainability [29, 43]. As a result, sustainable development needs constant access to clean, cost-efficient renewable energy sources that have no detrimental consequences [29].

Moreover, the ARDL/PMG estimates derived from the research conducted by Tweneboah-Koduah et al. affirm the role of renewable energy consumption as a driving force behind sustainable development [44]. This study also underscores the potential for renewable energy to yield substantial beneficial impacts in progressive nations. Additionally, in Germany, renewable energy is one of the significant contributions to the sustainable development of the country, therefore, the government should consider improving the share of renewable energy as policy instruments to achieve the sustainable goals [45]. Within the ASEAN region, the expansion of renewable energy plays a pivotal role in driving sustainable economic growth [46]. Furthermore, research conducted by Dincer (2000) has underscored the robust correlation between renewable energy and their utilization, and the promotion of sustainable development [47]. Hence, this study emphasizes the necessity for society to intensify its efforts in exploring sustainable energy sources, particularly renewable energy, as it strives to realize its sustainable development aspirations. Therefore, renewable energy development is essential for reducing the reliance on limited fossil fuel resources, mitigating pollution, and promoting a shift towards a more sustainable energy system, thus contributing to overall sustainable development. The following hypothesis is proposed:

***H3*: *The renewable energy of the environmental dimension of TBL has a positive effect on sustainable development*.**

## 3. Proposed model and econometric approach

### 3.1 Proposed model

In this study, the authors assess the hypothesis using quantitative models. Specifically, three core variables are examined: labor development, globalization, and renewable energy. Additionally, to ensure the robustness of the tests, the authors employ the model across various levels of development success and incorporate control variables.

The research is based on unbalanced panel data of 104 selected economies for the period from 2000 to 2020. This paper identified **the Failed group** are nations that have a decrease in rankings or continue low-income levels; countries with **lower middle** or **upper-middle Income Trap group** if those economies continue to maintain a low-middle or upper-middle income status, unable to transition to a high-income economy due to rising costs and reduced competitiveness. **Successful group** are countries that have increased to high-income levels or increased by two levels in the income group [21]. These economies are divided into groups depending on the growth of income level in S1 Appendix, including the failed income group, the low-middle income trap group, the high-middle income trap group, and the successful group.

To measure the *globalization* indicator, we used the KOF globalization index (LNKOFGI) provided by KOF Swiss Economic Institute from 2000 to 2020, a composite index measuring globalization for every country in the world along the economic, social and political dimension, which is measured on a scale from 1 to 100 [50]. Similarly, the labor factor is evaluated through the human development index (LNHDI) provided by the United Nations Development Programme (UNDP) from 2000 to 2020, on a scale from 0 to 1. According to UNDP, the human development index is a composite measure of average achievement in three key dimensions: health, education, and standard of living. It evaluates health by life expectancy at birth, education by the mean years of schooling for adults and expected years of schooling for children, and standard of living by gross national income per capita (adjusted logarithmically). The HDI is calculated as the geometric mean of these normalized indices, offering a comprehensive overview of human development. The renewable energy indicator is measured by the renewable energy (percentage of total final energy consumption) (LNREC), provided by the World Bank from 2000 to 2020. It is defined as the share of renewable energy in total final energy consumption. Besides, the sustainable development factor is estimated through the sustainable development goals scores (LNSDG), provided by the World Bank from 2000 to 2020. To calculate this index, the World Bank must estimate the score for each goal by using the arithmetic mean value of the indicators for that goal. These target scores are then averaged across all 17 SDGs to obtain the final index score.

In addition, we also use the employment rate (LNLABOR), the gross fixed capital formation (percentage of GDP) (LNKRATE), and political stability (LNPSI) as control variables. Namely, the employment rate (LNLABOR) provided by the World Bank from 2000 to 2020 is measured based on the employment rate of countries which is considered as employment to population ratio (15+, total % modeled ILO estimate). Gross fixed capital formation (percentage of GDP) (LNKRATE) comprises land improvements (fences, ditches, drains, etc.); plant, machinery, and equipment acquisitions; and the construction of roads, trains, and other structures, such as schools, offices, hospitals, private residential homes, and commercial and industrial buildings. Gross capital creation fixed percentage of GDP is established as a scale of this study since it can promote sustainable development. Data is taken from the World Bank starting from 2000 to 2020. Political stability (LNPSI), provided by the World Bank (https://databank.worldbank.org/source/world-development-indicators) from 2000 to 2020, is quantified based on the political stability and absence of violence/terrorism index which measures perceptions of the possibility of political unrest and/or politically motivated violence in units of the standard normal distribution, i.e. between -2.5 and 2.5.

### 3.2 Econometric approach

This paper applies long panel data models. Therefore, the authors employ panel unit root tests, co-integration tests, and estimate the effects of globalization, labor development, and renewable energy on SDG through the Pool Mean Group model.

#### Unit root tests

The use of unit root tests allows researchers to determine whether a sequence follows a random walk or reaches a stationary state, as a random walk implies non- stationarity. The importance of conducting a priori examination regarding the existence of unit roots in panel data arises from the recognized impact, whereby the presence of unit roots within the data could result in misinterpretations in the estimated results of methods such as the Least Squared Method (OLS), Generalized Least Squares (GLS), or the Pool Mean Group (PMG) model in the absence of data stationarity. To carry out this test, the simple correlation model is employed as follows:

$$\begin{array}{l} \gamma_{it}=\alpha_{i}+\gamma_{i,(t-1)}+\varepsilon_{it}\\ \gamma_{it}=\alpha_{it}+\sum_{k=1}^{t}\;\varepsilon_{it}+\gamma_{i,0}\\ \gamma_{it}=\alpha_{it}+y\gamma_{it} \end{array}$$

Where *γit* = *γi*,(*t*−1) + *uit* and the variable *γit* is entirely dependent on its string value, *γ*_*i*,(*t*−1)_. This paper utilizes the Im, Pesaran, and Shin (IPS) test in order to investigate the existence of unit roots. The IPS test, a panel unit root test, is widely preferred for its straightforward implementation and robustness to heterogeneity as it takes into account variations in autoregressive parameters across different models for individuals. According to Chen and Lu (2003), Im, Pesaran, and Shin’s panel unit root test (IPS) is also an extension of the individual ADF test [48]. It is formulated as a combination of independent unit root test statistics, employing the average of individual unit statistics to detect the presence of a unit root in panel data. Its usefulness is evident in empirical scenarios that involve a mixture of stationary and unit root series. Notably, it is applicable in examining the stationarity of exchange rates among different countries with varying exchange rate regimes, encompassing flexible rates with certain countries and fixed or semi-fixed rates with others.

#### Cointegration tests

Kao, and Kao & Chiang proposed panel tests for cointegration that can be seen as a generalization of the Dickey–Fuller (DF) and Augmented Dickey–Fuller (ADF) tests in the context of panel data [49, 50]. The tests involve setting the hypothesis of no cointegration as null, as well as constructing the test statistics and tabulating the distributions using the residuals derived from a panel static regression. Moreover, Kao and Pedroni tests assume that all relationships are either cointegrated or not cointegrated in the null and alternative hypotheses [49, 51, 52].

We investigate the power of five tests proposed, under different data- generating processes and when only a fraction of the relationships are really cointegrated [49]. Kao considered the spurious regression for the panel data and introduced the DF and ADF type tests [49]. The first two DF statistics assume strict exogeneity of the regressors with respect to the errors in the equation, while the other two allow for regressor endogeneity. The first is a Dickey–Fuller (DF) type test and the second is an Augmented- Dickey–Fuller (ADF) type test. Both tests can be calculated from:

$$\hat{u_{it}}=\rho \hat{u_{it-1}}+\sum_{j=1}^{\rho }\phi \hat{{\Delta u}_{it-j}}+v_{it}$$

The asymptotic distributions of all tests converge to a standard normal distribution N(0,1) as T → ∞ and N → ∞.

Despite considering both the time-series and cross-sectional dimension as aforementioned, many studies fail to reject the no-cointegration null, even when the theory strongly suggests cointegration. This is because most residual-based cointegration tests require the long-run parameters for the variables in their levels to be equal to the short-run parameters for the variables in their differences [52]. In response, Westerlund created four new panel cointegration tests that do not impose any common-factor restrictions, because they are based on structural dynamics rather than residual ones [52, 53]. The new tests are all normally distributed and are general enough to accommodate unit-specific short-run dynamics, unit-specific trend and slope parameters, and cross-sectional dependence.


$$\Delta y_{it}=\delta_{i}^{\prime }d_{t}+\alpha_{i}y_{i,t-1}+\lambda_{i}^{\prime }x_{i,t-1}+\sum_{j=1}^{\rho_{i}}\alpha_{ij}\Delta y_{i,t-j}+\sum_{j=-q_{i}}^{\rho_{i}}\gamma_{ij}\Delta x_{i,t-j}+e_{it}$$

The parameter αi determines the speed at which the system corrects back to the equilibrium relationship *y*_*i*,*t*−1_ – *β*′_*i*_*x*_*i*,*t*−1_ after a sudden shock. If *α*_*i*_ < 0, then there is error correction, which implies that *y*_*it*_ and *x*_*it*_ are cointegrated; if *α*_*i*_ = 0, then there is no error correction and, thus, no cointegration. Thus we can state the null hypothesis of no cointegration as *H*_0_: *α*_*i*_ = 0 for all i. The alternative hypothesis depends on what is being assumed about the homogeneity of *α*_*i*_. Two of the tests, called group-mean tests, do not require the *α*_*i*_*s* is to be equal, which means that *H*_0_ is tested versus $H_{1}^{g}$: *α*_*i*_ < 0 for at least one i. The second pair of tests, called panel tests, assume that *α*_*i*_*s* is equal for all i and are designed to test *H*_0_ versus $H_{1}^{g}$: *α*_*i*_ = *α* < 0 for all i.

#### Pool Mean Group (PMG)

This method enables distinct intercepts and short-run variable coefficients. Besides, the error variations vary across different groups of nations whereas the long-run regression coefficients are consistent across groups of countries. In other words, the PMG permits heterogeneity among the short-run estimations while pressuring the long-run parameters to be homogeneous. PMG offers a practical advantage to allow the various effects across the panel groups in compare to fixed-effect models. The PMG has many advantages over the conventional methods [54, 55]. Firstly, the error terms are not serially connected, in addition to being separately distributed among the regressors. Second, despite parameter homogeneity, the PMG generates reliable and effective long-term estimates. Thirdly, it keeps all cross-sectional units’ long-run properties constant. Fourthly, the PMG is particularly suitable when dealing with dynamic heterogeneous panels involving huge. Lastly, estimates produced by the PMG technique are also less susceptible to outliers. The PMG estimator is therefore considered to be the most suitable to investigate the relationship between variables in dynamic heterogeneous panel models. The PMG estimation is described as follows [55]:

$${\hat{\varphi }}_{PMG}=\frac{\sum_{i=1}^{N}{\hat{\varphi }}_{i}}{N}\;;\;{\hat{\beta }}_{PMG}=\frac{\sum_{i=1}^{N}{\hat{\beta }}_{i}}{N};$$

$${\hat{\lambda }}_{jPMG}=\frac{\sum_{i=1}^{N}{\hat{\lambda }}_{ij}}{N}\;,\;j=1,\ldots \ldots ,\rho -1$$

$${\hat{\Psi }}_{jPMG}=\frac{\sum_{i=1}^{N}{\hat{\Psi }}_{ij}}{N}\;,\;j=0,\ldots ..,q-1,$$

$$and\;{\hat{\theta }}_{PMG}=\hat{\theta }$$

PMG assumes identical long-run estimates of the model in the form ${\hat{\theta }}_{PMG}=\hat{\theta }$, and allows information from all countries in the sample to create a shared long-run estimate. This assumption can assist in reducing complexity in the analysis compared to examining the variation of long-run estimates for each country.

## 4. Results and discussion

### 4.1 Results

Descriptive statistics show a significant difference between the income group under study and the variables under examination (see Table 1). It presents summary statistics on the determinants of sustainable development, including the index measuring the main variables consisting of the KOF globalization index, human development index, renewable energy consumption, and control variables such as employment rate, gross fixed capital formation percentage of GDP, and political stability index. The highest average mean is observed for the SDG at 4.145987, indicating better performance or progress toward achieving sustainable development goals across the dataset. This is followed by the average mean of the KOF globalization index at 4.080724, reflecting the extent to which a country is integrated into the global economy, and indices related to gross fixed capital formation percentage of GDP (at 3.054851).

**Table 1:** Summary of descriptive statistics.

	LNSDG	LNKOFGI	LNHDI	LNREC	LNLABOR	LNKRATE	LNPSI
Min	3.621671	3.158772	-1.339411	-4.60517	-1.488539	.6933678	-8.469828
Mean	4.145987	4.080724	-.4058902	2.834656	-.8709275	3.054851	-.8014701
Max	4.458988	4.499295	-.0387408	4.564765	-.2981085	4.394709	.5614336
SD	.1796657	.2624576	.2663471	1.606784	.2220579	.3430462	1.165049
Skewness	-.6384216	-.4560186	-.8457705	-1.638332	-.5048695	-.9388318	-2.109341
Kurtosis	2.706947	2.730951	3.054365	6.747614	2.935543	8.061442	9.034704
Sum	9034.105	8891.897	-879.1583	6176.7150	-1897.751	6619.862	-730.9408

Regarding the maximum values, renewable energy consumption reveals the highest value (4.564765) within the dataset, suggesting a country with high renewable energy consumption that can lead to sustainable development for that specific data point. The subsequent indices followed by sustainable development goals scores, KOF globalization index, and gross fixed capital formation percentage of GDP, ranging from 4.394709 to 4.499295. In a similar vein, when considering variance, renewable energy consumption has the highest value at 1.606784, which is about 0.5 higher than the figure for the Political Stability Index (1.165049) and the corresponding figures in the remaining indexes are rather similar, from approximately 0.1796657 to 0.3430462.

The relationship between various determinants of sustainable development and their impact as measured by specific indices (see Table 2). It outlines the correlation between the dependent and the independent variables. All the independent variables, such as the KOF globalization index, human development index, renewable energy consumption, employment rate, gross fixed capital formation, and political stability index, exhibit positive correlations with sustainable development.

**Table 2:** Correlation matrix.

	LNSDG	LNKOFGI	LNHDI	LNREC	LNLABOR	LNKRATE	LNPSI
LNSDG	1.0000						
LNKOFGI	0.8579*	1.0000					
LNHDI	0.9334*	0.8919*	1.0000				
LNREC	-0.6675*	-0.6016*	-0.7298	1.0000			
LNLABOR	0.5395*	0.5791*	0.5942*	-0.2758*	1.0000		
LNKRATE	0.0704*	0.0142	0.0957*	-0.0637*	0.0790*	1.0000	
LNPSI	0.5778*	0.5766*	0.5995*	-0.2934*	0.5563*	0.1531*	1.0000

**Table 3** indicates that I(0) indicates that the original series is stationary, and I(1) means the first difference of a series is stationary. We show the results of the unit-root tests of the main variables SDG scores (LNSDG), KOF Globalization Index (LNKOFGI), employment rate, human development index (LNHDI), renewable energy consumption (LNREC), and the control variables including the employment rate (LNLABOR), gross fixed capital formation rate percentage of GDP (LNKRATE), and political stability index (LNPSI) to verify whether a time series variable is stationary or not.

**Table 3:** Unit root tests.

Variable	I(0)	I(1)
LNSDG	21.0316	-26.1185***
LNKOFGI	-6.3545***	-22.9047***
LNHDI	-4.9882***	-11.0642***
LNLABOR	4.9174	-11.6793***
LNKRATE	-1.1640	-18.8991***
LNREC	9.0861	-21.6522***
LNPSI	-7.5121***	-25.6830***

Note

*,**,*** are significance at 10%, 5%, 1% respectively.

The results show that three variables, namely the KOF globalization index, human development index, and political stability index, do not have unit roots. This means that the series of these variables is stationary at a 1% significance level. The null hypothesis (*H*_0_), which assumes that all panels contain unit roots, was rejected. Meanwhile, the other variables, namely SDG, renewable energy consumption, employment rate, and gross fixed capital formation rate (% of GDP) have unit roots. Thus, their series is not yet stationary. In order to transform them into a stationary time series, we continue to use a differencing process. After taking the first difference I(1), all of the time series become stationary at a 1% significance level. This means that the variables share common trend and may have a long-term relationship with each other.

In summary, it is important to take the assumption of stationary data into consideration in time series analysis, as many time series analysis approaches rely on the underlying patterns and relationships to remain unchanged across time. The test results from Table 3 show that all time series data becomes stationary at the first difference I(1) at a significance level of 1%. As a consequence, the relationship between sustainable development and other variables has been able to be verified in the following steps.

Following the execution of panel unit root tests, we proceed with conducting panel cointegration analysis in **Table 4**. The long-term relationship between variables was examined using the Kao and Westerlund tests [49, 53].

**Table 4:** Cointegration tests.

	Kao Test	Westerlund Test
Modified Dickey–Fuller t	3.7161***	Some panels: -1.677 *** All Panel: -1.324 *
Dickey–Fuller t	4.7812***	
Augmented Dickey–Fuller t	5.0633***	
Unadjusted modified Dickey–Fuller	2.9183***	
Unadjusted Dickey–Fuller t	3.9462***	

According to the outcomes derived in the Kao and Westerlund panel cointegration tests, the null hypothesis (which assumes the absence of cointegration between the series) was denied in all statistics at the 1% significance level. Thus, it indicates a notable cointegration relationship among the variables.

According to the outcomes derived in the Kao panel cointegration test, the null hypothesis (which assumes the absence of cointegration between the series) was denied in all statistics at the 1% significance level. Thus, it indicates a notable cointegration relationship among the variables [49]. To tackle the limitations of the Kao test, an alternative test from the second generation of panel cointegration tests, known as the Westerlund test, was employed. It is an error-correction-based panel cointegration test and is recognized for its enhanced robustness since it internally handles structural breaks and cross-sectional dependence concerns [53]. The Westerlund test results indicated substantial values for certain panels and all panel statistics, with their corresponding robust p-values being significant at the 1% and 10% levels, respectively. Consequently, the *H*_0_ hypothesis, suggesting no cointegration, was rejected. Therefore, the study accepted the alternative hypothesis proposing the existence of cointegration.

In brief, the Kao test results indicate strong cointegration among the relevant variables at a significance level of 1%. This implies long-term interactions among the panel data series. Additionally, the bootstrap cointegration test by Westerlund further supports this finding [53], showing significant cointegration at both the 1% and 10% levels, respectively. To conclude, there is substantial evidence supporting the long-term relationship between sustainable development and other variables, aligning with the findings in the previous studies [19, 20, 44].

**Table 5** represents the results of the impact of independent and control variables on SDG scores in each country’s group with different levels of income based on the PMG model.

**Table 5:** Pool Mean Group (PMG).

	All	FailedGroup	LowMiddleTrap	HighMiddleTrap	Success
LNKOFGI	-0.0518***	-0.0717**	0.458***	-0.0971**	0.0737***
	(-3.38)	(-2.03)	(6.45)	(-2.07)	(3.28)
LNHDI	77.82***	82.35***	24.59***	48.16***	38.65***
	(40.69)	(18.81)	(3.31)	(9.90)	(7.76)
LNREC	0.0171***	0.0460**	-0.0565	0.00653	0.337***
	(3.32)	(2.25)	(-0.78)	(0.17)	(11.87)
LNLABOR	19.37***	28.87***	-5.635	35.81***	-0.260
	(10.15)	(2.93)	(-0.45)	(7.54)	(-0.06)
LNKRATE	-0.0142***	-0.00883	-0.125***	0.0776**	0.119***
	(-3.96)	(-1.52)	(-2.86)	(2.09)	(4.26)
LNPSI	-0.128	-0.135	-0.710*	0.723**	0.00676
	(-1.63)	(-1.10)	(-1.79)	(2.03)	(0.04)
**Model Summary**					
R2					
Pesaran CD	63.29%	64.80%	62.96%	64.79%	62.35%
Z statistic	8 (0.00)	-0.07 (0.9)	-0.7 (0.5)	0.8 (0.4)	4 (0.00)
(pvalue)					

Note

*,**,*** are significance at 10%, 5%, 1% respectively.

There are different results of how globalization impacts on sustainable development of countries’ groups with different levels of income. KOF globalization index (KOFGI) has a negative impact on the sustainable development of all countries at the 1% significance level. Specifically, when KOFGI increases by 1%, SDG scores of this group will decrease by 5,18%. Similarly, KOFGI also has a negative impact on the failed group and high-middle income trap group nations at the 10% significance level, specifically, when KOFGI increases by 1%, SDG scores will decrease by 7,17% for the failed group and decrease by 9,71% for the high-middle income trap group. Conversely, KOFGI has a positive influence on the economies in the low- middle income trap group and successful group at the 1% significance level, the SDG scores of these two groups of countries will increase respectively by 45,8% and 7,37% when KOFGI increases by 1%.

Meanwhile, the human development index (HDI) has a positive impact on all countries with different levels of income development at the 1% significance level. Specifically, if HDI increases by 1%, SDG scores of all countries increase by 7782%, the failed group increases by 8235%, the low-middle income trap group increases by 2459%, the high-middle income trap group increases by 4816% and the successful group increases by 3865%.

Renewable energy consumption (REC) also has different impacts on the SDG Score of different country groups. Between REC and SDG scores of all countries generally and the successful group in particular, there is a positive impact from REC on the sustainable development of these economies at the 1% significance level, in which, if REC increases by 1%, the SDG scores of the group of all countries and the successful group will increase respectively by 1,71% and 33,7%. Similarly, REC also has a positive effect on the failed group at the 5% significance level, meaning that its SDG scores will increase by 4,6% when REC increases by 1%. However, for the low-middle income trap group and high-middle income trap group, the results suggest that the impact of REC is not significant for these two groups.

Regarding employment rate (LABOR), the results demonstrate that LABOR only has a positive impact on SDG scores of all countries generally, especially in the failed group and high-middle income trap group in particular at the 1% significance level. When LABOR increases by 1%, the SDG Scores of these groups also increase by 1937%, 2887%, and 3581%, respectively. LABOR also does not have an influence on several countries in the low-middle income trap group and successful group.

The gross fixed capital formation percentage of GDP (KRATE) has different impacts on SDG scores of all groups of countries, except the failed group. In particular, KRATE has a negative impact on the sustainable development of all countries and low-middle income trap group at the 1% significance level, in which, when KRATE increases by 1%, the SDG scores of these groups decrease respectively by 1,42% and 12,5%. KRATE also has a positive impact on the high-middle income trap group at the 5% significance level, specifically SDG Score will increase by 7,76% when KRATE increases by 1%.

Finally, the political stability index (PSI) only has an impact on the low-middle income trap group and the high-middle income trap group. For low-middle income economies, PSI has a negative impact at the 10% significance level, meaning that if PSI increases by 1%, the SDG scores of this group decrease by 71%. On the contrary, PSI has a positive impact at the 5% significance level on the high-middle income trap group. Specifically, SDG Scores of this group will increase by 72,3% when PSI increases by 1%.

### 4.2 Discussion

This study proves that globalization has a complex influence on the sustainable development of various country groups. On one hand, globalization has a positive impact on sustainable development in low-middle and successful group countries. It provides opportunities for trade, investment, and technological transfer, which can contribute to economic growth and sustainable development. Countries that can capitalize on these opportunities have experienced rapid economic growth. Moreover, transitioning from middle- income to high-income is more challenging and can be hindered by the high middle-income trap. The high middle-income trap refers to the difficulty that many countries face in sustaining their economic growth and transitioning to high-income status. Several factors, such as market expansion, technological advancements, infrastructure development, and demographic dividends, have been identified as crucial for overcoming the middle-income trap and achieving high-income status [54, 55]. Therefore, while globalization can facilitate the transition from low to middle-income, additional efforts and strategies are needed to overcome the challenges of the upper middle-income trap and achieve high-income status [8, 20, 56]. Studies show a positive link between globalization and sustainable development, especially for countries with strong economic performance (as measured by trade and finance). Benefits include short-term access to financial aid, knowledge, and green technologies, and long-term positive impacts on sustainability.

On the other hand, our study also shows that globalization has a negative influence on sustainable development in certain countries within the failed group and high-middle group economies. These perspectives share the same view of paper for panel data of South Asian economics from 1985 to 2018 which reveals a negative association between globalization and sustainable development indicators such as income, health, and education [57]. Similarly, Mishkin states that while globalization can foster economic growth by removing trade barriers and facilitating the free flow of goods and services, it may also hinder sustainable development in certain contexts [58]. This means the distribution process of globalization is often inefficient, leading to economic disparities and increased vulnerability to global economic crises. In terms of sustainability, globalization has both positive and negative impacts. It facilitates the exchange of goods and services across borders, but it also accelerates the uncontrolled exploitation of natural resources, leading to environmental degradation by driving increased industrial activity and resource exploitation [15, 16, 20, 21]. As countries become more integrated into the global economy, they often intensify the use of natural resources to meet international demand, leading to deforestation, pollution, and loss of biodiversity. Moreover, globalization is also associated with a rise in income inequality by disproportionately benefiting wealthier nations and individuals. While it can stimulate growth, the benefits are unevenly distributed, often leaving poorer countries and local industries struggling to compete [59]. This disparity is further compounded by the exploitation of labor in developing regions, where low wages and poor working conditions are common. Wealth becomes concentrated among multinational corporations and elites, widening the income gap and increasing economic inequality both within and between countries.

Moreover, the labor factor emerges as a crucial determinant of sustainable development across all economies. The positive impact of the human development index, which encompasses health, education, and standard of living dimensions in econometric results, suggests that all groups of countries are committed to addressing the social and labor implications of sustainable development. Labor’s income share, which measures the share of national income earned by labor, is an important aspect of sustainable development and an equitable society. This finding is consistent with the studies confirmed that continued efforts to enhance labor productivity and reduce unemployment can lead to improved social stability and higher living standards for the general population [39, 41]. More specifically, enhanced labor productivity means that workers are more efficient and can produce more output per hour worked, which can lead to higher wages and better living standards. Reduced unemployment not only increases individual income but also contributes to overall economic stability. These factors together improve the human development index— encompassing health, education, and living standards—highlighting that investments in labor are essential for achieving sustainable development goals and ensuring broad-based economic and social benefits.

The paper’s findings reveal the significance of renewable energy as a key dimension of sustainable development in several countries. The utilization of renewable energy sources derived from natural resources, including geothermal, hydroelectric, tidal, biomass, wind, solar, and biofuels, has been demonstrated to foster sustainable development. By looking at the positive effect of renewable energy on sustainable development in many group countries, it is evident that both the failed-income and high-success income economies recognize the crucial role of renewable energy in shaping their environment. These high-success economies, without a doubt, are deeply concerned about the environmental implications of renewable energy for human well-being and the preservation of biodiversity. By prioritizing the consumption of renewable energy over non-renewable sources (e.g. fossil fuels), they can avoid the shortage of limited non-renewable energy supply in the future and reduce energy emissions. Meanwhile, the group of countries with the potential to establish a state of renewable energy industrialization, despite holding substantial fossil fuel reserves (e.g. Mozambique, Sudan, Syrian Arab Republic, Chad, and Uganda), can capitalize on the abundant renewable energy resources available in nature. Additionally, economies with limited non-renewable fuel sources (e.g. Burundi, Burkina Faso, Central African Republic, Guinea, Gambia, Madagascar, Niger, Rwanda, and Togo) also promote renewable energy to ensure energy security, curtail energy pollutants, and pre-empt future energy shortages. These findings are consistent with the conclusions of some studies, such as OECD countries [19], 24 sub-Saharan African countries (SSA) [44], or ASEAN countries [46]. These studies persistently highlight renewable energy as a leading driver of sustainable development, emphasizing its contribution to increasing the share of renewable energy in total final energy consumption.

Generally, the impact of the globalization factor on sustainable development varies across groups of countries. Both groups with low incomes and high middle trap incomes, experience more pronouncedly negative effects compared to the overall. Conversely, two groups having high incomes and a low middle trap income, are positively impacted. Notably, sustainable development in the group with a low middle trap income tends to increase more significantly than in the high-income nations when the globalization factor decreases, and vice versa. Generally, all groups of countries benefit positively from the labor factor. Specifically, in the case of an increase in this factor, countries with low incomes demonstrate the most robust sustainable development compared to other country groups. Subsequently, ranking in descending order of influence, these groups are countries with a high middle income trap, high income, and finally, with low middle income trap respectively. The renewable energy factor has different impacts on the sustainable development of each income group of countries. Firstly, considering positive impacts, the high-income group is most strongly affected, the lower affected ones the low-income group, and lastly, the high middle trap income countries, which experiences the least positive impact compared to all countries. Conversely, nations with a low intermediate income trap face a negative influence from the renewable energy factor. However, this impact is not excessively high.

## 5. Conclusion and implications

This paper has analyzed the factors that determine sustainable development in a sample of 104 selected economies using unbalanced panel data for the period from 2000 to 2020. In order to accomplish the paper’s purpose, we utilize both the panel unit root and the panel cointegration tests. Besides, the error variances that differ between distinct groupings of nations are measured by using the panel PMG estimation. Our main evidence on the positive influence of labor on sustainable development is also consistent with the positive impact of the HDI index. The results reveal that labor and renewable energy have a positive impact on sustainable development in nations with decreasing rankings or persistently low incomes. Conversely, globalization has a negative effect in these cases. In lower middle-income trap countries, globalization and labor positively influence sustainable development, while renewable energy has a negative impact. In the while, upper- middle-income trap nations face adverse effects from globalization but benefit from labor and renewable energy factors in sustainable development. Finally, countries with high incomes or a significant increase in income levels experience positive influences from all three factors (labor, renewable energy, and globalization) on sustainable development.

Our findings highlight that the labor factor has a positive impact on the sustainable development of all groups of economies. Thereby, the importance of educational policies is even more clearly affirmed in developing a sustainable economy. The government needs to focus more on improving the quality of education in countries, updating and adjusting all teaching-learning and assessment methods at schools based on the advanced education systems in the world. However, in practice, when implementing these policies, the biggest obstacle in many countries, particularly developing and underdeveloped nations, is the capital required to invest in educational projects. Firstly, there is a shortage of financial resources to improve infrastructure such as schools, learning materials, and research facilities. Additionally, there is a scarcity of human capital, including high-quality teachers and highly skilled advisors in the field, which remains a significant challenge for the education systems of these countries. Furthermore, these nations often lack opportunities to access advanced methods, techniques, and technologies in the education sector to apply and enhance the quality of education domestically. Therefore, to effectively upgrade the quality of education in these countries, the government should first implement policies to attract investment into educational projects from domestic private enterprises and foreign investors to address the budget shortfalls in education. Additionally, to enhance the competency of human resources in the education sector, the government should enact policies that encourage self-improvement among teachers and academic advisors. This can be achieved by utilizing the funds raised from businesses and organizations to expand professional development courses for teachers and offer scholarships for overseas study to individuals in this field who have demonstrated excellence in teaching.

Regarding empirical findings, we find that globalization has a positive influence on sustainable development for low-middle and high-success income nations. In contrast, the KOF globalization index observed from the failed and high-middle countries negatively affects sustainable development. Additionally, in all nations, the labor factor positively affects sustainable development. Our primary evidence on the positive impact of the human development index implies that all groups of nations are dedicated to tackling the social and labor issues of sustainable development. Therefore, improving social stability and raising the standard of living for the general public is essential, and this can be achieved by reducing unemployment and raising labor productivity. Reducing the unemployment rate and enhancing labor productivity still face numerous challenges when applied in practice. Firstly, reducing the unemployment rate in countries is heavily dependent on the current economic situation. With the development and application of AI technology in production and business to increase efficiency and reduce costs, many companies globally have laid off hundreds, even thousands, of employees. Consequently, this leads to a decrease in consumer spending, negatively impacting the economy and contributing to the current recession. Therefore, to effectively reduce unemployment and increase labor productivity, governments need to implement policies that improve the skills and qualifications of the workforce to adapt to domestic and global economic development trends. They must also mitigate the negative impacts of replacing human activities with AI technology in various fields. Specifically, governments should encourage workers to enhance their knowledge and skills through promotional activities within enterprises, companies, and factories. Additionally, policymakers should enact decrees and actions to diversify the economy, promote the development of new sectors, and apply advanced technologies smartly and efficiently to avoid over-reliance on modern technology. The results show that globalization has both positive and negative impacts on sustainable development. In particular, globalization has a positive impact on the sustainable development of economies in the low-middle income trap group and successful group. Hence, the governments of these countries should focus on policies that promote integration into world economic trends and expand the scale of globalization not only in the economy but also in other fields. In terms of foreign policies, it is important that the policies should stimulate bilateral-multilateral trade, investment, the establishment of economic alliances as they can eliminate trade barriers, and increase the connection between supply chains of countries around the world. However, expanding connections with other countries may face limitations if the government does not make appropriate decisions. Promoting international trade can increase competitive pressure on domestic products, leading to the dominance of foreign enterprises and negatively affecting the domestic economy. Moreover, if not tightly controlled, allowing external resources to enter the country can result in excessive dependence on other nations, causing the government and citizens to gradually lose their autonomy and fall into the traps of dangerous global forces. To avoid these consequences, the government needs to accurately and objectively assess the national situation, then carefully consider and select diplomatic directions. After successfully establishing relationships with other countries, the government should implement policies to promote the development of domestic products and avoid monopolies. Additionally, it is essential to strengthen and sustainably develop national resources to limit dependence on foreign investment.

Renewable energy consumption is a key dimension of sustainable development. In line with our findings, the utilization of renewable energy sources can foster sustainable development in both the failed-income and high-success-income economies group. It can be argued that if high-success economies go for broad usage of renewable energy rather than non- renewable sources in their consumption and production activities, there is an opportunity to lower energy emissions and prevent future shortages of the finite supply of non-renewable energy. In addition, economies with finite supplies of non-renewable fuels are encouraged to use renewable energy to reduce energy pollution, guarantee energy security, and anticipate future energy shortages. Although countries are currently striving to reduce non-renewable energy consumption, significant obstacles remain. The primary barrier is the initial investment cost for renewable energy technologies such as wind, solar, and marine energy, which require substantial resources and capital. The existing distribution and storage systems are predominantly designed for non-renewable energy, necessitating significant upgrades and expansions to infrastructure, which involve considerable time and expense. Renewable energy also heavily depends on weather and geographical conditions, such as solar power relying on sunlight and wind power on wind speed, making it challenging to maintain a stable supply. For developing countries, policies supporting renewable energy development are often incomplete, lacking clarity and stability, which poses difficulties for investors and businesses. Additionally, the awareness of the importance of renewable energy among the public and businesses is limited. Therefore, governments need to implement educational and communication programs to raise awareness and encourage the use of renewable energy. Countries can also collaborate to build networks and infrastructure for renewable energy production through international organizations and agreements, helping to overcome financial, technological, and resource barriers. The consumption of renewable energy also has a positive effect on the sustainable development of several countries in the failed group and successful group. Depending on the situation of each economy, the government should have appropriate solutions to adjust energy consumption in the country. In the case of these two groups of countries, increasing use of renewable energy has partly helped promote sustainable development. Thus, policies can be applied as follows: encouraging businesses and households to switch from using non-renewable energy to using natural energy such as solar energy, wind energy, etc. The government can support businesses that are planning to convert and also businesses that are selling products and services related to renewable energy to help reduce costs for both parties, increasing the number of consumers.

## Supporting information

S1 Appendix (DOCX)
