# Correction: The impact of globalization, renewable energy, and labor on sustainable development: A cross-country analysis

**DOI:** 10.1371/journal.pone.0333199

**Published:** 2025-09-24

**Authors:** Huynh Ngoc Chuong, Vo Tran Phuong Uyen, Nguyen Dang Phuong Ngan, Nguyen Thi Bao Tram, Nguyen Dao Mai Han, Pham Hoang Khanh Duyen

The affiliation for this article is incorrect. The correct affiliation is as follows,

University of Economics and Law, Ho Chi Minh City, Vietnam and Vietnam National University, Ho Chi Minh City, Vietnam.
