# Supplementary material for: The impact of globalization, renewable energy, and labor on sustainable development: A cross-country analysis
Source: PLoS One. 2025 Feb 21;20(2):e0315273. doi: 10.1371/journal.pone.0315273 (PMC11844918; doi:10.1371/journal.pone.0315273)
Supplement: S1 Appendix — (DOCX) [file pone.0315273.s001.docx]

**Appendix**

**List of 104 economies divided into groups with different levels of development from 2000 to 2020.**

| **No.** | **Failed Group** | **Low Middle Income Trap Group** | **High Middle**  **Income Trap Group** | **Successful Group** |
| --- | --- | --- | --- | --- |
| 1 | Burundi | Algeria | Argentina | Angola |
| 2 | Burkina Faso | Egypt, Arab Rep. | Brazil | Benin |
| 3 | Central African  Republic | Iran, Islamic Rep. | Botswana | Bangladesh |
| 4 | Guinea | Morocco | Costa Rica | Bulgaria |
| 5 | Gambia, The | In Swat | Gabon | Bhutan |
| 6 | Madagascar | Tunisia | Mexico | Cote d'Ivoire |
| 7 | Mozambique |  | Montenegro | Cameroon |
| 8 | Niger |  | Mauritius | Congo, Rep. |
| 9 | Rwanda |  | Malaysia | Comoros |
| 10 | Sudan |  | Serbia | Cuba |
| 11 | Syrian Arab Republic |  | Türkiye | Dominican  Republic |
| 12 | Chad |  | South Africa | Ecuador |
| 13 | Togo |  | United Arab  Emirates | Fiji |
| 14 | Uganda |  | Australia | Guatemala |
| 15 |  |  | Austria | Indonesia |
| 16 |  |  | Barbados | India |
| 17 |  |  | Canada | Iraq |
| 18 |  |  | Cyprus | Jamaica |
| 19 |  |  | Germany | Jordan |
| 20 |  |  | Denmark | Kazakhstan |
| 21 |  |  | Spain | Kenya |
| 22 |  |  | Finland | Kyrgyz Republic |
| 23 |  |  | France | North  Macedonia |
| 24 |  |  | United Kingdom | Mongolia |
| 25 |  |  | Greece | Mauritania |
| 26 |  |  | Israel | Namibia |
| 27 |  |  | Italy | Nigeria |
| 28 |  |  | Japan | Nicaragua |
| 29 |  |  | Norway | Pakistan |
| 30 |  |  | Portugal | Peru |

| 31 |  |  | Singapore | Paraguay |
| --- | --- | --- | --- | --- |
| 32 |  |  | Slovenia | Russian  Federation |
| 33 |  |  | Sweden | Senegal |
| 34 |  |  | United States | Tajikistan |
| 35 |  |  |  | Tanzania |
| 36 |  |  |  | Ukraine |
| 37 |  |  |  | Uzbekistan |
| 38 |  |  |  | Vietnam |
| 39 |  |  |  | Zimbabwe |
| 40 |  |  |  | Armenia |
| 41 |  |  |  | Azerbaijan |
| 42 |  |  |  | Czechia |
| 43 |  |  |  | Estonia |
| 44 |  |  |  | Georgia |
| 45 |  |  |  | Korea, Rep. |
| 46 |  |  |  | Moldavia |
| 47 |  |  |  | Panama |
| 48 |  |  |  | Saudi Arabia |
| 49 |  |  |  | Slovak Republic |
| 50 |  |  |  | Uruguay |
